# 3-Bromo-*N*′-(3,5-dibromo-2-hydroxy­benzyl­idene)benzohydrazide methanol solvate

**DOI:** 10.1107/S1600536809007466

**Published:** 2009-03-06

**Authors:** Yi-Jun Wei, Feng-Wu Wang, Qi-Yong Zhu

**Affiliations:** a Department of Chemistry, Huainan Normal College, Huainan 232001, People’s Republic of China

## Abstract

The title compound, C_14_H_9_Br_3_N_2_O_2_·CH_4_O, was prepared by the reaction of 3,5-dibromo-2-hydroxy­benzaldehyde and 3-bromo­benzohydrazide in methanol. The asymmetric unit of the crystal consists of a Schiff base mol­ecule and a methanol mol­ecule of crystallization. The dihedral angle between the two benzene rings is 5.5 (2)°. An intra­molecular O—H⋯N hydrogen bond is observed. In the crystal structure, pairs of adjacent Schiff base mol­ecules are linked by two methanol mol­ecules through inter­molecular N—H⋯O and O—H⋯O hydrogen bonds.

## Related literature

For the synthesis of Schiff bases, see: Annigeri *et al.* (2002 ▶); Lodeiro *et al.* (2003 ▶); Rao *et al.* (2003 ▶). For related structures, see: Bao & Wei (2008 ▶); Odabaşoğlu *et al.* (2007 ▶); Wang *et al.* (2006 ▶); Wei *et al.* (2008 ▶); Yathirajan *et al.* (2007 ▶); Yehye *et al.* (2008 ▶); Zhu *et al.* (2009 ▶).
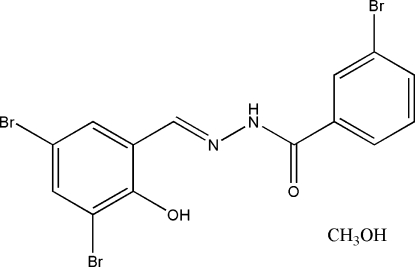


## Experimental

### 

#### Crystal data

C_14_H_9_Br_3_N_2_O_2_·CH_4_O
*M* *_r_* = 509.00
Triclinic, 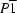

*a* = 8.900 (1) Å
*b* = 9.366 (1) Å
*c* = 11.392 (2) Å
α = 95.043 (2)°
β = 111.048 (2)°
γ = 99.584 (2)°
*V* = 862.6 (2) Å^3^
*Z* = 2
Mo *K*α radiation
μ = 7.03 mm^−1^
*T* = 298 K
0.23 × 0.20 × 0.20 mm

#### Data collection

Bruker SMART 1000 CCD area-detector diffractometer
Absorption correction: multi-scan (*SADABS*; Sheldrick, 1996 ▶) *T*_min_ = 0.216, *T*_max_ = 0.245
5016 measured reflections
3606 independent reflections
2582 reflections with *I* > 2σ(*I*)
*R*_int_ = 0.019

#### Refinement

*R*[*F* ^2^ > 2σ(*F* ^2^)] = 0.035
*wR*(*F* ^2^) = 0.084
*S* = 1.03
3606 reflections
214 parameters
1 restraint
H atoms treated by a mixture of independent and constrained refinement
Δρ_max_ = 0.45 e Å^−3^
Δρ_min_ = −0.61 e Å^−3^

### 

Data collection: *SMART* (Bruker, 2002 ▶); cell refinement: *SAINT* (Bruker, 2002 ▶); data reduction: *SAINT*; program(s) used to solve structure: *SHELXS97* (Sheldrick, 2008 ▶); program(s) used to refine structure: *SHELXL97* (Sheldrick, 2008 ▶); molecular graphics: *SHELXTL* (Sheldrick, 2008 ▶); software used to prepare material for publication: *SHELXTL*.

## Supplementary Material

Crystal structure: contains datablocks global, I. DOI: 10.1107/S1600536809007466/sj2587sup1.cif

Structure factors: contains datablocks I. DOI: 10.1107/S1600536809007466/sj2587Isup2.hkl

Additional supplementary materials:  crystallographic information; 3D view; checkCIF report

## Figures and Tables

**Table 1:** Hydrogen-bond geometry (Å, °)

*D*—H⋯*A*	*D*—H	H⋯*A*	*D*⋯*A*	*D*—H⋯*A*
O1—H1⋯N1	0.82	1.84	2.559 (3)	145
O3—H3⋯O2^i^	0.82	1.97	2.767 (4)	164
N2—H2⋯O3^ii^	0.90 (3)	1.986 (18)	2.848 (4)	160 (4)

Symmetry codes: (i) 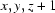; (ii) 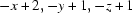.

**Fig. 1.**
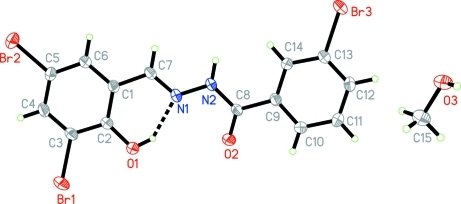
The molecular structure of the title compound, showing 30% probability displacement ellipsoids. The dashed line indicates an intramolecular hydrogen bond.

**Fig. 2.**
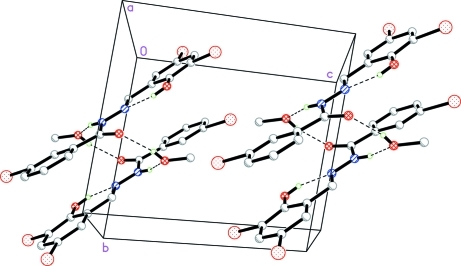
Molecular packing of the title compound. Hydrogen bonds are shown as dashed lines.

## supplementary crystallographic information

### Comment

Schiff bases are readily synthesized by the reaction of aldehydes with primary amines (Lodeiro *et al.*, 2003; Annigeri *et al.*, 2002; Rao *et al.*, 2003). We have previously reported some Schiff bases and their complexes (Wei *et al.*, 2008; Wang *et al.*, 2006). In this paper, the preparation and crystal structure of the new Schiff base title  compound (I), Fig 1, is reported.

The C═N bond length in the title molecule is comparable with those observed in other Schiff bases (Yehye *et al.*, 2008; Odabaşoğlu *et al.*, 2007; Yathirajan *et al.*, 2007). All bond lengths are within normal ranges and are comparable to those observed in the related compounds (Zhu *et al.*, 2009; Bao & Wei, 2008). The dihedral angle between C1—C6 and C9—C14 phenyl rings is 5.5 (2)°, indicating that the molecule is nearly coplanar. An intramolecular O1—H1···N1 hydrogen bond is observed and may contribute to the overall planarity of the molecule.

In the crystal structure, pairs of adjacent Schiff base molecules are linked by two methanol molecules through intermolecular N2—H2···O3 and O3—H3···O2 hydrogen bonds, Table 1, Fig. 2.

### Experimental

3,5-Dibromo-2-hydroxybenzaldehyde (1.0 mmol) and 3-bromobenzohydrazide (1.0 mmol) were dissolved in methanol (30 ml). The mixture was stirred at reflux for 10 min to give a clear colourless solution. After keeping this solution in air for a week, colourless block-shaped crystals were formed.

### Refinement

The  H atom bound to N2 was located in a difference Fourier map and refined isotropically, with the N—H distance restrained to 0.90 (1) Å. All other H atoms were positioned geometrically (C—H = 0.93-0.96 Å and O—H = 0.82 Å) and refined as riding, with *U*_iso_(H) values set at 1.2*U*_eq_(C) and 1.5*U*_eq_(O and C15). The crystals were small and weakly diffracting which accounts for the low measured data fraction of 96% out to θ = 27.0 °.

### Crystal data

**Table:**

C_14_H_9_Br_3_N_2_O_2_·CH_4_O	*Z* = 2
*M**_r_* = 509.00	*F*(000) = 492
Triclinic, *P*1	*D*_x_ = 1.960 Mg m^−^^3^
Hall symbol: -P 1	Mo *K*α radiation, λ = 0.71073 Å
*a* = 8.900 (1) Å	Cell parameters from 1965 reflections
*b* = 9.366 (1) Å	θ = 2.5–28.0°
*c* = 11.392 (2) Å	µ = 7.03 mm^−^^1^
α = 95.043 (2)°	*T* = 298 K
β = 111.048 (2)°	Block, colorless
γ = 99.584 (2)°	0.23 × 0.20 × 0.20 mm
*V* = 862.6 (2) Å^3^	

### Data collection

**Table:**

Bruker SMART 1000 CCD area-detector diffractometer	3606 independent reflections
Radiation source: fine-focus sealed tube	2582 reflections with *I* > 2σ(*I*)
graphite	*R*_int_ = 0.019
ω scans	θ_max_ = 27.0°, θ_min_ = 1.9°
Absorption correction: multi-scan (*SADABS*; Sheldrick, 1996)	*h* = −11→10
*T*_min_ = 0.216, *T*_max_ = 0.245	*k* = −11→11
5016 measured reflections	*l* = −12→14

### Refinement

**Table:**

Refinement on *F*^2^	Primary atom site location: structure-invariant direct methods
Least-squares matrix: full	Secondary atom site location: difference Fourier map
*R*[*F*^2^ > 2σ(*F*^2^)] = 0.035	Hydrogen site location: inferred from neighbouring sites
*wR*(*F*^2^) = 0.084	H atoms treated by a mixture of independent and constrained refinement
*S* = 1.03	*w* = 1/[σ^2^(*F*_o_^2^) + (0.0368*P*)^2^ + 0.2522*P*] where *P* = (*F*_o_^2^ + 2*F*_c_^2^)/3
3606 reflections	(Δ/σ)_max_ = 0.001
214 parameters	Δρ_max_ = 0.45 e Å^−^^3^
1 restraint	Δρ_min_ = −0.61 e Å^−^^3^

### Special details

**Table:**

Geometry. All esds (except the esd in the dihedral angle between two l.s. planes) are estimated using the full covariance matrix. The cell esds are taken into account individually in the estimation of esds in distances, angles and torsion angles; correlations between esds in cell parameters are only used when they are defined by crystal symmetry. An approximate (isotropic) treatment of cell esds is used for estimating esds involving l.s. planes.
Refinement. Refinement of F^2^ against ALL reflections. The weighted R-factor wR and goodness of fit S are based on F^2^, conventional R-factors R are based on F, with F set to zero for negative F^2^. The threshold expression of F^2^ > 2sigma(F^2^) is used only for calculating R-factors(gt) etc. and is not relevant to the choice of reflections for refinement. R-factors based on F^2^ are statistically about twice as large as those based on F, and R- factors based on ALL data will be even larger.

### Fractional atomic coordinates and isotropic or equivalent isotropic displacement parameters (Å^2^)

**Table:**

	*x*	*y*	*z*	*U*_iso_*/*U*_eq_	
Br1	0.70334 (5)	1.00574 (5)	−0.37638 (4)	0.07063 (16)	
Br2	1.39490 (6)	1.16831 (6)	−0.18075 (5)	0.08319 (19)	
Br3	1.13101 (5)	0.31103 (4)	0.49994 (3)	0.05564 (13)	
O1	0.7708 (3)	0.8094 (3)	−0.1804 (2)	0.0524 (6)	
H1	0.7891	0.7546	−0.1269	0.079*	
O2	0.6880 (3)	0.5145 (3)	−0.0140 (2)	0.0564 (7)	
O3	0.7124 (3)	0.4000 (3)	0.7628 (2)	0.0634 (7)	
H3	0.6886	0.4388	0.8192	0.095*	
N1	0.9495 (3)	0.7027 (3)	0.0069 (2)	0.0402 (6)	
N2	0.9553 (4)	0.6172 (3)	0.0991 (3)	0.0410 (6)	
C1	1.0672 (4)	0.8813 (3)	−0.0850 (3)	0.0351 (7)	
C2	0.9137 (4)	0.8887 (4)	−0.1748 (3)	0.0378 (7)	
C3	0.9096 (4)	0.9860 (4)	−0.2607 (3)	0.0425 (8)	
C4	1.0505 (4)	1.0671 (4)	−0.2633 (3)	0.0449 (8)	
H4	1.0450	1.1288	−0.3236	0.054*	
C5	1.2002 (4)	1.0569 (4)	−0.1762 (3)	0.0460 (8)	
C6	1.2101 (4)	0.9668 (4)	−0.0856 (3)	0.0424 (8)	
H6	1.3121	0.9632	−0.0251	0.051*	
C7	1.0795 (4)	0.7865 (3)	0.0117 (3)	0.0389 (8)	
H7	1.1807	0.7877	0.0755	0.047*	
C8	0.8112 (4)	0.5247 (4)	0.0818 (3)	0.0388 (7)	
C9	0.8101 (4)	0.4374 (3)	0.1852 (3)	0.0365 (7)	
C10	0.6573 (4)	0.3690 (4)	0.1800 (3)	0.0487 (9)	
H10	0.5620	0.3809	0.1160	0.058*	
C11	0.6468 (5)	0.2827 (5)	0.2705 (4)	0.0607 (11)	
H11	0.5442	0.2361	0.2670	0.073*	
C12	0.7878 (5)	0.2654 (4)	0.3657 (4)	0.0538 (10)	
H12	0.7811	0.2077	0.4268	0.065*	
C13	0.9373 (4)	0.3341 (4)	0.3692 (3)	0.0407 (8)	
C14	0.9518 (4)	0.4212 (3)	0.2807 (3)	0.0375 (7)	
H14	1.0550	0.4679	0.2853	0.045*	
C15	0.5947 (6)	0.4070 (6)	0.6437 (4)	0.0784 (14)	
H15A	0.5748	0.5045	0.6420	0.118*	
H15B	0.4939	0.3389	0.6291	0.118*	
H15C	0.6346	0.3822	0.5784	0.118*	
H2	1.054 (3)	0.617 (5)	0.159 (3)	0.080*	

### Atomic displacement parameters (Å^2^)

**Table:**

	*U*^11^	*U*^22^	*U*^33^	*U*^12^	*U*^13^	*U*^23^
Br1	0.0520 (3)	0.0897 (3)	0.0683 (3)	0.0262 (2)	0.0085 (2)	0.0444 (2)
Br2	0.0529 (3)	0.0928 (4)	0.0939 (4)	−0.0098 (2)	0.0193 (2)	0.0505 (3)
Br3	0.0528 (2)	0.0679 (3)	0.0421 (2)	0.01579 (19)	0.00793 (17)	0.02572 (18)
O1	0.0349 (13)	0.0634 (17)	0.0562 (15)	0.0067 (11)	0.0111 (12)	0.0302 (13)
O2	0.0364 (14)	0.0847 (19)	0.0458 (14)	0.0112 (13)	0.0087 (12)	0.0325 (13)
O3	0.0423 (15)	0.087 (2)	0.0534 (15)	0.0155 (14)	0.0067 (13)	0.0188 (15)
N1	0.0430 (16)	0.0410 (16)	0.0396 (14)	0.0114 (13)	0.0152 (13)	0.0199 (12)
N2	0.0402 (16)	0.0463 (16)	0.0387 (15)	0.0110 (13)	0.0132 (13)	0.0220 (13)
C1	0.0383 (18)	0.0363 (17)	0.0315 (16)	0.0076 (14)	0.0131 (14)	0.0099 (13)
C2	0.0372 (18)	0.0398 (18)	0.0363 (17)	0.0108 (15)	0.0117 (15)	0.0102 (14)
C3	0.042 (2)	0.047 (2)	0.0378 (18)	0.0150 (16)	0.0109 (16)	0.0151 (15)
C4	0.052 (2)	0.043 (2)	0.0396 (18)	0.0100 (17)	0.0146 (17)	0.0175 (15)
C5	0.044 (2)	0.044 (2)	0.049 (2)	0.0008 (16)	0.0177 (17)	0.0164 (16)
C6	0.0371 (18)	0.0434 (19)	0.0422 (18)	0.0056 (15)	0.0094 (15)	0.0138 (15)
C7	0.0406 (19)	0.0391 (19)	0.0357 (17)	0.0119 (15)	0.0094 (15)	0.0144 (14)
C8	0.0369 (18)	0.0457 (19)	0.0398 (18)	0.0155 (15)	0.0159 (16)	0.0195 (15)
C9	0.0392 (18)	0.0391 (18)	0.0346 (16)	0.0115 (14)	0.0151 (15)	0.0124 (14)
C10	0.0339 (19)	0.061 (2)	0.054 (2)	0.0124 (17)	0.0155 (17)	0.0246 (18)
C11	0.042 (2)	0.077 (3)	0.073 (3)	0.0102 (19)	0.028 (2)	0.039 (2)
C12	0.052 (2)	0.062 (2)	0.059 (2)	0.0154 (19)	0.0278 (19)	0.0336 (19)
C13	0.0411 (19)	0.046 (2)	0.0342 (16)	0.0104 (16)	0.0113 (15)	0.0130 (15)
C14	0.0329 (17)	0.0427 (19)	0.0374 (17)	0.0066 (14)	0.0135 (14)	0.0118 (14)
C15	0.052 (3)	0.115 (4)	0.056 (2)	0.010 (3)	0.005 (2)	0.034 (3)

### Geometric parameters (Å, °)

**Table:**

Br1—C3	1.886 (3)	C4—H4	0.9300
Br2—C5	1.889 (3)	C5—C6	1.378 (4)
Br3—C13	1.891 (3)	C6—H6	0.9300
O1—C2	1.339 (4)	C7—H7	0.9300
O1—H1	0.8200	C8—C9	1.494 (4)
O2—C8	1.221 (4)	C9—C14	1.381 (4)
O3—C15	1.400 (4)	C9—C10	1.381 (5)
O3—H3	0.8200	C10—C11	1.385 (5)
N1—C7	1.265 (4)	C10—H10	0.9300
N1—N2	1.367 (3)	C11—C12	1.378 (5)
N2—C8	1.361 (4)	C11—H11	0.9300
N2—H2	0.90 (3)	C12—C13	1.364 (5)
C1—C6	1.388 (5)	C12—H12	0.9300
C1—C2	1.399 (4)	C13—C14	1.378 (4)
C1—C7	1.461 (4)	C14—H14	0.9300
C2—C3	1.390 (4)	C15—H15A	0.9600
C3—C4	1.365 (5)	C15—H15B	0.9600
C4—C5	1.370 (5)	C15—H15C	0.9600
			
C2—O1—H1	109.5	O2—C8—N2	121.2 (3)
C15—O3—H3	109.5	O2—C8—C9	121.5 (3)
C7—N1—N2	120.2 (3)	N2—C8—C9	117.2 (3)
C8—N2—N1	115.8 (3)	C14—C9—C10	120.4 (3)
C8—N2—H2	125 (3)	C14—C9—C8	123.2 (3)
N1—N2—H2	118 (3)	C10—C9—C8	116.4 (3)
C6—C1—C2	120.0 (3)	C9—C10—C11	119.6 (3)
C6—C1—C7	119.1 (3)	C9—C10—H10	120.2
C2—C1—C7	120.8 (3)	C11—C10—H10	120.2
O1—C2—C3	118.5 (3)	C12—C11—C10	120.3 (3)
O1—C2—C1	123.4 (3)	C12—C11—H11	119.8
C3—C2—C1	118.1 (3)	C10—C11—H11	119.8
C4—C3—C2	121.7 (3)	C13—C12—C11	119.0 (3)
C4—C3—Br1	119.5 (2)	C13—C12—H12	120.5
C2—C3—Br1	118.8 (3)	C11—C12—H12	120.5
C3—C4—C5	119.5 (3)	C12—C13—C14	122.0 (3)
C3—C4—H4	120.2	C12—C13—Br3	119.2 (3)
C5—C4—H4	120.2	C14—C13—Br3	118.8 (2)
C4—C5—C6	120.8 (3)	C13—C14—C9	118.6 (3)
C4—C5—Br2	119.4 (3)	C13—C14—H14	120.7
C6—C5—Br2	119.8 (3)	C9—C14—H14	120.7
C5—C6—C1	119.7 (3)	O3—C15—H15A	109.5
C5—C6—H6	120.2	O3—C15—H15B	109.5
C1—C6—H6	120.2	H15A—C15—H15B	109.5
N1—C7—C1	118.6 (3)	O3—C15—H15C	109.5
N1—C7—H7	120.7	H15A—C15—H15C	109.5
C1—C7—H7	120.7	H15B—C15—H15C	109.5

### Hydrogen-bond geometry (Å, °)

**Table:**

*D*—H···*A*	*D*—H	H···*A*	*D*···*A*	*D*—H···*A*
O1—H1···N1	0.82	1.84	2.559 (3)	145
O3—H3···O2^i^	0.82	1.97	2.767 (4)	164
N2—H2···O3^ii^	0.90 (3)	1.99 (2)	2.848 (4)	160 (4)

Symmetry codes: (i) *x*, *y*, *z*+1; (ii) −*x*+2, −*y*+1, −*z*+1.

## References

[bb1] Annigeri, S. M., Naik, A. D., Gangadharmath, U. B., Revankar, V. K. & Mahale, V. B. (2002). *Transition Met. Chem.* **27**, 316–320.

[bb2] Bao, X. & Wei, Y.-J. (2008). *Acta Cryst.* E**64**, o1682. 10.1107/S160053680802360X PMC2960532 21201672

[bb3] Bruker (2002). *SMART *and *SAINT* Bruker AXS Inc., Madison, Wisconsin, USA.

[bb4] Lodeiro, C., Bastida, R., Bértolo, E., Macías, A. & Rodríguez, A. (2003). *Transition Met. Chem.* **28**, 388–394.

[bb5] Odabaşoğlu, M., Büyükgüngör, O., Narayana, B., Vijesh, A. M. & Yathirajan, H. S. (2007). *Acta Cryst.* E**63**, o1916–o1918.

[bb6] Rao, P. V., Rao, C. P., Wegelius, E. K. & Rissanen, K. (2003). *J. Chem. Crystallogr.* **33**, 139–147.

[bb7] Sheldrick, G. M. (1996). *SADABS* University of Göttingen, Germany.

[bb8] Sheldrick, G. M. (2008). *Acta Cryst.* A**64**, 112–122. 10.1107/S0108767307043930 18156677

[bb9] Wang, F.-W., Wei, Y.-J. & Zhu, Q.-Y. (2006). *Chin. J. Struct. Chem.* **25**, 1179–1182.

[bb10] Wei, Y.-J., Wang, F.-W. & Zhu, Q.-Y. (2008). *Transition Met. Chem.* **33**, 543–546.

[bb11] Yathirajan, H. S., Vijesh, A. M., Narayana, B., Sarojini, B. K. & Bolte, M. (2007). *Acta Cryst.* E**63**, o936–o938.

[bb12] Yehye, W. A., Ariffin, A. & Ng, S. W. (2008). *Acta Cryst.* E**64**, o1452. 10.1107/S1600536808020746 PMC2962083 21203167

[bb13] Zhu, C.-G., Wei, Y.-J. & Zhu, Q.-Y. (2009). *Acta Cryst.* E**65**, o85.

